# Multifaceted Interplay among Social Dominance, Body Condition, Appetitive and Consummatory Sexual Behaviors, and Semen Quality in Dorper Rams during Out-Of-Season and Transition Periods

**DOI:** 10.3390/ani12233339

**Published:** 2022-11-29

**Authors:** Pablo I. Sifuentes-Lamónt, Cesar A. Meza-Herrera, Francisco G. Véliz-Deras, Alan S. Alvarado-Espino, Ariadna V. Alvarado-Espino, Guadalupe Calderón-Leyva, Oscar Angel-Garcia, Dalia I. Carrillo-Moreno, Viridiana Contreras-Villarreal, Ramón A. Delgado González, Jorge A. Bustamante-Andrade

**Affiliations:** 1 Universidad Autónoma Agraria Antonio Narro, Unidad Laguna, Periférico Raúl López Sánchez y Carretera a Santa Fe, Torreon 27054, Coahuila, Mexico; 2 Universidad Autónoma Chapingo, Unidad Regional Universitaria de Zonas Áridas, Bermejillo, 35230, Durango, Mexico; 3 Facultad de Agricultura y Zootecnia, Universidad Juárez del Estado de Durango, Gómez Palacio 35111, Durango, Mexico

**Keywords:** sheep, social hierarchy, reproductive fitness, hemogram

## Abstract

**Simple Summary:**

The effect of social rank (i.e., low or high) in adult Dorper rams on sexual behavior, body condition score, seminal quality, and whole blood count was evaluated. The high-social-ranked Dorper rams displayed more sexual behaviors compared with low-social-ranked rams. In parallel, high-social-ranked rams had a higher body condition score and a larger ejaculate volume compared with low-social-ranked rams; they also showed an optimal health and wellness status, reflected by the whole blood count. Such behavioral, metabolic, sexual, seminal, and health advantages shown by the high-social-ranked Dorper rams must be taken into account to avoid reproductive failures. While there is still limited evidence on the role that social rank plays in out-of-season reproductive success, this study helps to better comprehend how social dominance is associated with male-to-male competitiveness, male-to-female sexual behavior, seminal quality, and blood cell components, and how their interaction with each other modulates and even determines out-of-season reproductive success in Dorper rams.

**Abstract:**

Dorper rams (*n* = 24) were evaluated during the sexual resting season to determine their social rank (SR), either high (HSR) or low (LSR), under intensive management conditions in northern Mexico (25° N). Aggressive behaviors were quantified during male-to-male interactions, and appetitive and consummatory sexual behaviors during male-to-female interactions. Morphometric, live weight (LW), and body condition score (BCS) were recorded. During the early reproductive season, male-to-female behaviors were newly itemized simultaneously by seminal quality and quantity sampling. Finally, the dependent variables of the hemogram components were also quantified. Neither LW (61.25 ± 2.4 kg) nor morphometric variables differed between SR groups. However, BCS (2.25 vs. 2.66 u), sexual behaviors (i.e., approaches: 59.6 vs. 21.73 n, mating with ejaculation: 77.7 vs. 42.86 %, latency to ejaculation: 16.6 vs. 143.07 s), ejaculate volume (0.57 vs. 0.23 mL), and hemogram components favored the HSR rams (*p* < 0.05). Moreover, in their first male-to-female interaction, >50% of the LSR rams failed to display any sexual activity. HSR rams displayed a greater number of threatening behaviors, managing to displace LSR rams when exposed to estrus ewes during the male sexual resting season; more sexual behaviors; and an increased seminal volume in a non-live weight-dependent fashion.

## 1. Introduction

Domestic and wild small ruminants are gregarious species that live in long-lasting and solid social groups. Sheep, in general, privilege such gregarious behavior in order to lower the risk of predation [1,2]. The social hierarchy system is one of the most outstanding peculiarities among these gregarious species, under any type of social organization [3]. A social hierarchy is defined as a range of individuals, in a social unit, centered on reciprocal relationships of dominance–subordination; the more aggressive the animal, the higher the social rank [4]. In fact, low-social-rank animals protect themselves by not physically instigating high-ranking animals in obtaining resources and trying to reduce their exposure to physical damage [5]. Living in social groups provides their members with the benefit of increasing learning opportunities, especially linked to the eating behaviors of the group’s companions. This learning can optimize a strategy to achieve more time available for feeding, greater probability of access to individuals of the opposite gender, and a higher survival rate, in both sheep [6] and goats [4]. Under natural conditions, the availability of different resources is a determining factor in the formation of a group. In domestic animals, life in social groups is determined by the different types of management systems [6]. Moreover, the relations of dominance and subordination among animals also modulate the productive and reproductive outcomes in male and females. In fact, high-social-rank females have a greater opportunity to interact with males compared to those of low social rank [7]. Additionally, ewes of high social rank (HSR) display a significant relationship between social dominance and reproductive success [1].

In sheep [8] and goats [9], most of the breeds located in temperate or subtropical latitudes of the world have developed seasonal breeding patterns, which ensure that late gestation and lactation are prevented during spring and summer. In this way, the probabilities of survival, both for the mother and the offspring, are increased by the combination of moderate temperatures and abundant grass [8,9]. The photoperiod is the foremost environmental factor that determines reproductive seasonality [10]. Because of this, in the sexual activity season males have increased levels of reproductive hormone concentrations, such as testosterone [8,10].

Regarding hierarchy, high-social-rank (HSR) rams show a greater number of appetitive and consummatory sexual behaviors than low-social-rank (LSR) rams; however, said sexual behavior is highly correlated to the live weight of males [11]. Moreover, the importance of live weight for reproductive success is greater for males than for females [1]. The hematological parameters of male sheep vary depending on the production system (e.g., extensive, semi-intensive, and intensive) but not the seasons of the year (e.g., winter and summer) [12]. However, to our knowledge there is no evidence on the association between blood components and social rank.

The foregoing supports the fact that social rank is positively related to a differential access to available resources, granting in turn better productive and reproductive outcomes for HSR males. Nevertheless, under intensive production schemes, feeding areas, feeders, and even feed distribution are significantly optimized to improve equal access to available resources among animals, as well as to limit dominance–subordination scenarios. Hence, we hypothesized that in Dorper rams managed under an intensive production system, differences in live weight may exist, yet other factors such as morphometry, body condition, and some plasma constituents defining health status will engender a social hierarchy strategy ensuring both improved sexual behavior and semen quality in HSR rams, both in the reproductive resting season and the early reproductive season. Therefore, this study was designed to answer these questions and to test such a working hypothesis.

## 2. Materials and Methods

### 2.1. Location, Environment, Commercial Herd, Experimental Groups, and General Management

**Figure 1:**
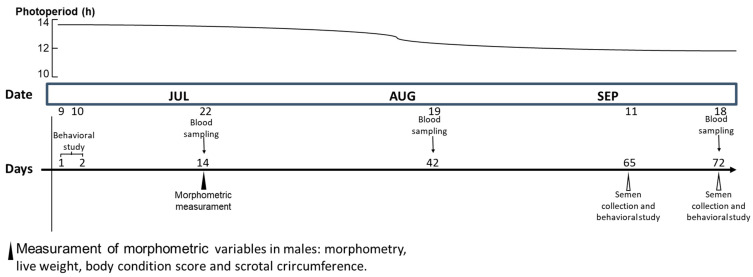
Timeline of the main activities completed in the experimental period. We conducted a behavioral study in all the experimental units (*n* = 24) to determine the Dorper males’ social rank, either high or low, under intensive management conditions in northern Mexico (25° N) during a photoperiod of decreasing long days (13:41–12:00). In early July, the behavioral test (male-to-male and male-to-female) was carried out in the morning (0800 h × 60 min, two days). In late September, the rams’ height at the withers, length, thoracic perimeter, live weight, body condition score, and scrotal circumference were recorded. In mid-September, two semen collections and a second male-to-female sexual behavioral test were performed. Monthly blood sampling was performed (July, August, and September) to build a hemogram. Throughout the complete experimental trial, there was no indication of any health-related issues in any of the experimental units.

The present experiment was carried out in a meat sheep intensive production farm (open corrals) located in northern Mexico (Comarca Lagunera, 25°35′ N, 103°17′ W, 1120 m). The general herd comprised 1000 ewes and 50 rams belonging to the breeds Dorper (90%) and Black Belly (10%). The Comarca Lagunera is classified as a semi-arid ecotype, with an average annual temperature of 22 °C with lows of −5 °C (winter) and highs of 45 °C (summer). The photoperiod fluctuates from 13 h 41 min at the summer solstice to 10 h 19 min at the winter solstice [13]. Regarding the experimental groups, Dorper rams (*n* = 24; 3 ± 0.5 year) were kept in individual open pens (3 × 4 m) while ewes were housed in groups of 50 in open pens (15 × 20 m); all sheep were managed under the same intensive management and environmental conditions. Each ram received 2.8 kg of a food mixture (corn, stubble, mineral salts, molasses, and cotton hulls), 1.4 kg in the morning (0800 h) and 1.4 kg in the afternoon (1800 h), designed to cover their nutritional requirements [14], and clean water was provided *ad libitum.* All animals received fat-soluble vitamins (A: 500,000 UI; D: 75,000 UI; E: 50 UI) and were dewormed one month prior to the experimental period. Sanitation practices to control the fly population were carried in both the general corrals and the experimental pens. The handling of male and female sheep was in agreement with guidelines for the ethical use, care, and welfare of animals in research at both international [15] and national [16] levels, with reference institutional consent number UAAAN-UL-21-2840. The photoperiod and chronology of the main activities performed through the experimental period are shown in Figure 1.

### 2.2. Quantifying the Agonistic Behavioral Activity during the Sexual Resting Season

During the sexual resting season (i.e., early July), a paired behavioral test was carried out to determine the social rank (i.e., high or low) of each male (Figure 1). The first behavioral study started at 0800 h; the main agonistic behavior between two rams (i.e., male-to-male interactions) in the presence of one estrus female were registered. To determine the social rank of each male, rams were exposed to both Black Belly and Dorper ewes (*n* = 24; 3.5 ± 0.5 year); ewes were distributed in two groups (*n* = 12) and were placed in individual pens (2.25 × 2.25 m). The behavioral test was carried out over 2 days; on the first day, the first group of ewes (*n* = 12) was exposed to the 24 males in a paired fashion, and the second set of ewes (*n* = 12) was used during the second day. Each pair of males was observed simultaneously during 3 min trials when exposed to the estrus ewes (i.e., 2 males × 1 female × 3 min × pen). At the end of the three minutes, one of the males was moved to the next female pen. In the following three minutes, the male that had remained in the pen during the previous test was moved to the next pen in a sequential fashion. Therefore, each one of the males had interaction with the other 23 males and with a different female; consequently, a total of 23 behavioral tests were performed on each male. This paired-ram arrangement allowed for competition between a male and the rest of the males under study, thus ensuring we did not repeat or omit any possible combination of males during the behavioral tests. The agonistic social behaviors and the male-to-female sexual interactions were recorded by 12 people trained for this specific and crucial task.

The recorded agonistic behaviors considered included: (1) threats, when a male tried to hit another male; the threat could be made with the head or the trunk of the body, but without contact; (2) hitting, when a male hit another individual with his head; and (3) pushing, when a male pushed another male with his body, but without hitting him, in order to move him from a certain place. Taking into account the behavior records, the success rate (SI) was calculated for each male [4]. The SI shows the relationship of agonistic interactions that culminate when an animal displaces another animal, that is, when the male wins the interaction. An individual that wins all of its interactions has an SI of 1, whereas an animal losing all of its interactions has an IE of 0. With the SI obtained, the males were classified into two social ranks: rams with an SI from 0 to 0.5 were classified in the low social rank; rams with an SI greater than 0.5 and up to 1 were classified in the high social rank. Then, a success index (SI) was obtained from each experimental unit by using the formula:

$$\mathrm{SI}=\frac{\mathrm{number}\;\mathrm{of}\;\mathrm{won}\;\mathrm{events}}{\mathrm{number}\;\mathrm{of}\;\mathrm{won}\;\mathrm{events}\;+\;\mathrm{number}\;\mathrm{of}\;\mathrm{lost}\;\mathrm{events}}$$

### 2.3. Quantifying Rams’ Morphometric Variables, Live Weight, and Body Condition Score

**Figure 2:**
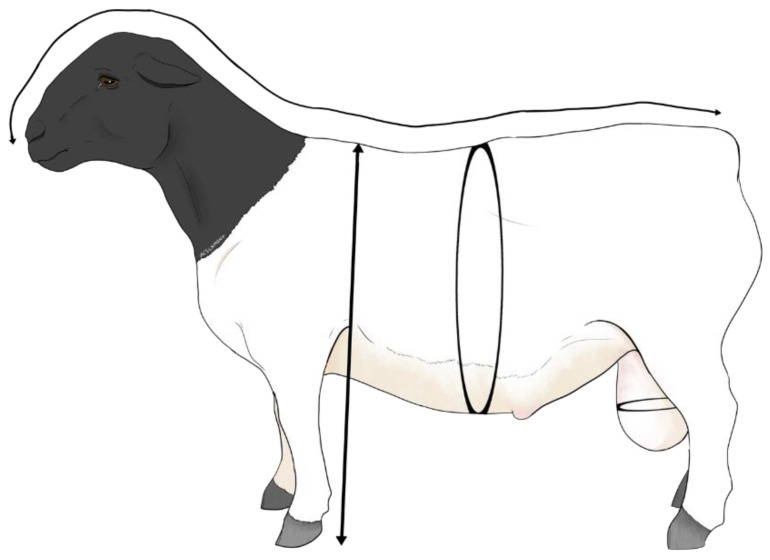
Morphological variables evaluated with a flexible plastic tape, and their body reference points: scrotal circumference (SCRC, cm), the tape was placed around the wider part of the scrotum; height at the withers (HEIG, cm), from the hoof to the withers; length (LENG, cm) from the snout to the base of the tail; and thoracic perimeter (PERI, cm), around the ribs, according to both low- and high-social-rank Dorper rams (*n* = 24) in northern Mexico (25° N).

During the sexual resting season, 12 days after the sexual behavioral test (Figure 1), morphometric variables were evaluated. Besides live weight (LW, kg) and body condition score (BCS, units), the morphometric variables of height at the withers (HEIG, cm), body length (LENG, cm), thoracic perimeter (PERI, cm), and scrotal circumference (SCRC, cm) were registered. Live weight was determined using an electronic scale with a precision of 50 g and a capacity of 250 kg (Torrey 110v/220v, Digital Industrial Scale, Jalisco, Mexico). In addition, BCS was measured by a qualified technician by palpating the transverse processes of the lumbar vertebrae [17], with a scale of 1 (emaciated) to 5 (obese). To measure the scrotal circumference and morphometry, a flexible plastic tape measure was used, graduated every millimeter. The specific body landmark for each variable is shown in Figure 2.

### 2.4. Quantifying the Sexual Male-to-Female Behavior during the Sexual Resting Season

In the course of the sexual resting season (i.e., July), we recorded the first agonistic male-to-male behaviors for each ram (i.e., 3 min × 2 days), and the sexual behavior of males displayed when having contact with one estrus ewe was also recorded (Figure 1). Later, in the reproductive season (i.e., September), a second male-to-female behavior recording was carried out (Figure 1). On the first day of each behavioral study, the male rejection to mount the estrus ewes was assumed to be an indicator of sexual inactivity. Both the appetitive and consummatory sexual behaviors of rams were recorded when they had contact with the estrus female. The registered sexual behaviors were: (1) approaches, (2) flehmen, (3) anogenital sniffing, and (4) mounts, as previously outlined [18]. An approach was defined as the male approaching the female with a brief sound emission and an outstretched foreleg movement toward the female, the flehmen reflex as the particular behavior of the male turning his upper lip upwards, an anogenital sniff as the male bringing his nose closer to the female’s anogenital area, and mounting as the male-to-female interaction after a period of locomotion, during which the male had an erection and then performed an overlay on the female.

### 2.5. Quantifying Rams’ Seminal Quality during the during the Early Reproductive Season

In September, during the early reproductive season, the males were exposed again (one day weekly at 0800 h) to estrus females in order to collect semen with an artificial vagina (Figure 1). Then, macro- and microscopic evaluations were performed, registering the following seminal response variables: sperm concentration (×10⁶/mL), mass motility (i.e., 0–5 u), and ejaculated volume (mL). Sperm concentration was measured using the photometer SDM 1 calibrated for ovine semen (Minitube^®^, Tiefenbach, Germany), and mass motility was measured using the phase-contrast microscope Olympus CX43 (Minitube^®^, trinocular and heated stage, Tiefenbach, Germany). Furthermore, the number of matings with ejaculation and ejaculation latency (i.e., seconds), when rams had contact with the estrus female, were registered as consummatory behaviors, as previously defined [18].

### 2.6. Plasmatic Hemogram Analyses

Over the entire experimental period, all rams were blood sampled to determine blood count values (Figure 1). Samples were collected by jugular venipuncture and placed in 4 mL BD Vacutainer^®^ tubes (Broken Bow, NE, USA) containing 7.2 mg of K2 EDTA, a coagulation activator. Subsequently, the samples were used to quantify complete blood count by an automated cell counter, Hemalyzer 1000 (Minneapolis, MN, USA), performed by a trained technician. The complete blood count included the total count of leukocytes, recording the result in units of 10^9^ cells per liter (×10^9^/L); the total count of erythrocytes, in units of 10^12^ cells per liter (×10^12^/L); the amount of hemoglobin, an oxygen-carrying protein found inside erythrocytes, in grams per deciliter (g/dL); the hematocrit, which measures the proportion (%) of the total blood volume that consists of erythrocytes; the mean corpuscular volume, which indicates the average size of erythrocytes and is expressed in femtoliters (fL); the mean corpuscular hemoglobin, the average amount of hemoglobin (pg) within each erythrocyte; the mean corpuscular hemoglobin concentration, a calculated measure of how concentrated the hemoglobin is within the erythrocytes (g/dL); and erythrocyte distribution width, a measure of the variation (%) in the size of erythrocytes [19].

### 2.7. Statistical Analyses

To quantify the possible association of social rank status with the corporal variables (i.e., LW, kg, BCS, units, and morphometric variables), linear models were carried out, and the fixed effect of social rank status and the residual error were considered. Then, due to the fact that both percentage and count variables did not fit normal distributions, they were log^10^ transformed to reduce skewness (i.e., different components of the hemogram) prior to ANOVA. All the sexual behaviors, as well as hemogram component data, were analyzed by means of a mixed linear model (PROC MIXED). In the final model, the fixed effect was the social rank whereas the repeated measurement was the sampling date; the random effect was the ram’s ID within his social rank. Since social rank was individually determined, each ram was considered an experimental unit. Least squares means and standard errors for each class of social rank status, time, and the interaction between these two factors were calculated and used for multiple mean comparisons using Fisher’s least significant difference with the LSMEANS option of SAS. All the analyses were performed through the procedures of SAS (SAS Inst. Inc., Version 9.4, Cary, NC, USA). Statistical differences between mean values were set at *p* ≤ 0.05.

## 3. Results

### 3.1. Behavioral Male-to Male Interactions during the Sexual Resting Season

The male-to-male behaviors recorded in all rams during the sexual resting season, in order to define the social hierarchy, are shown in Table 1. The number of threatening behaviors was higher in the HSR (3.22 ± 0.7) than in the LSR (0.86 ± 0.5) group (*p* = 0.011). The number of hitting and pushing behaviors did not differ between the HSR and LSR groups (*p* > 0.05).

### 3.2. Ram Morphometric Variables, Body Weight, Body Condition Score, and Scrotal Circumference

The morphometric variables LW, BCS, and SCRC evaluated in male Dorper sheep are shown in Table 2. While the HSR group showed the highest BCS (*p* < 0.05), no differences (*p* > 0.05) occurred between hierarchies for LW and SCRC. Table 2 also includes the morphometric values of HEIG, LENG, and thoracic perimeter PERI; no differences (*p* > 0.05) arose between hierarchies.

### 3.3. Simple Effects of Social Rank and Time upon Behavioral Male-to-Female Activities

The indicators of the appetitive and consummatory sexual behaviors, recorded in all rams during both the resting and early reproductive activity seasons (i.e., July and Sept, respectively) are shown in Table 3. The HSR group had the best sexual performance with the lowest values for the rejection of mating (%) compared to the LSR males. The number of approaching behaviors was higher in the HSR than LSR group (*p* = 0.0001). Moreover, the number of mounting behaviors was higher in the HSR than LSR group (*p* = 0.0001).

### 3.4. Interaction of Social Rank by Time Effects upon Behavioral Male-to-Female Activities

**Figure 3:**
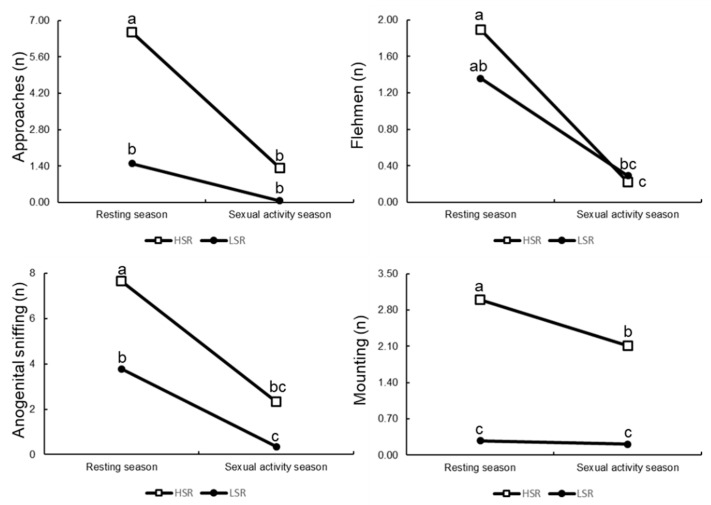
Least squares means for the appetitive and consummatory sexual behaviors, considering approaches, flehmen, anogenital sniffing, and mounting, according to either low (*n* = 15) or high (*n* = 9) social rank in Dorper rams in northern Mexico (25° N), during resting and sexual activity season. ^a,b,c^ Least squares means lacking a mutual superscript within response variable differ (*p* < 0.05).

The indicators of the appetitive and consummatory sexual behaviors, recorded in all rams during both the resting and early reproductive activity seasons (i.e., July and Sept) are shown in Figure 3/Table 4. As observed, all the response variables were affected by the SR x T interaction (*p* = 0.001). Interestingly, the LSR group showed the highest rejection to mate (%), as compared to the HSR males (*p* = 0.001); thus, HSR rams had a higher number of consummatory behaviors (i.e., mounting) in both seasons (i.e., July and Sept).

### 3.5. Consummatory Behaviors and Indicators of Semen Quality during the Early Reproductive Season

The indicators of the consummatory sexual behaviors, and some indicators of semen quality during the early reproductive activity season (i.e., Sept), are shown in Table 5. The HSR group had the best sexual performance with the lowest values for latency to ejaculation(s), as compared to the LSR males. Regarding seminal quality, the HSR group also exhibited the best indicators regarding the response variables of mating with ejaculation (%) and ejaculated volume (mL). No differences were observed between social hierarchies regarding the response variables of sperm concentration (×10⁶/mL) and mass motility (u) (*p* > 0.05). Table 5 shows the values regarding the number of sexual behaviors of males when exposed to direct contact with ewes in estrus.

### 3.6. Simple Effects of Social Rank & Time on Some Blood Plasma Constituents (i.e., Hemogram)

The hemogram dependent variables of erythrocytes (ERYTH, ×10^12^/L), hemoglobin (HB, g/dL), hematocrit (HT, %), mean corpuscular volume (MCV, fL), and erythrocyte distribution width (EDW, %), according to the simple effects of social rank and time, are shown in Table 6. Since the response variables of leukocytes (LEUKO, ×10⁹/L), mean corpuscular hemoglobin (MCHB, pg), and mean corpuscular hemoglobin concentration (MCHBC, g/dL) were affected by the SR *x* T interaction, these independent variables are not included in Table 6. No differences were observed between ERYTH, HB, HT, MCV, and EDW regarding social rank (*p* > 0.05) or time of collection (i.e., either reproductive arrest or early reproductive activity).

### 3.7. Interaction of Social Rank by Time upon Some Blood Plasma Constituents in Dorper Rams

**Figure 4:**
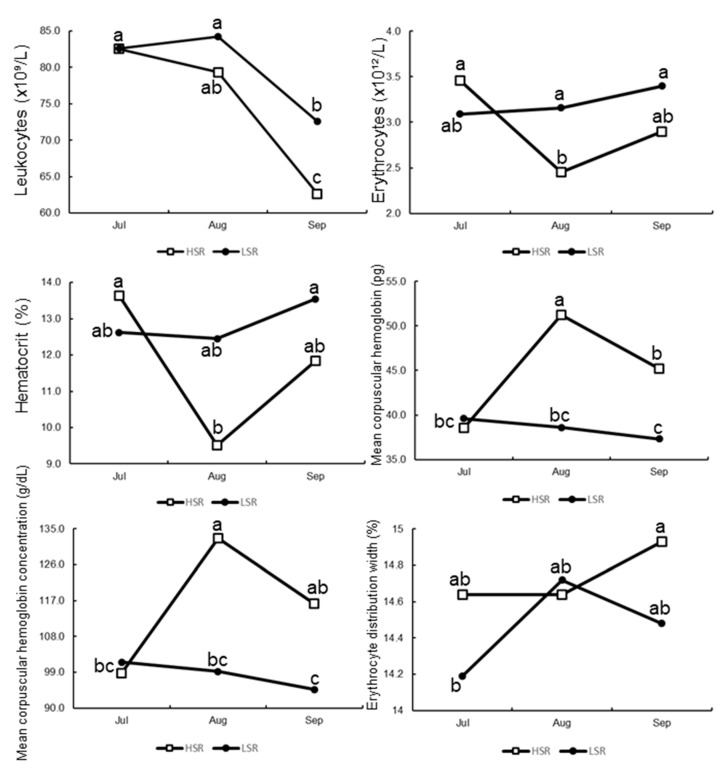
Least square means for leucocytes (×10⁹/L), erythrocytes (×10^12^/L), hematocrit (%), mean corpuscular hemoglobin (pg), mean corpuscular hemoglobin concentration (mg/dL), and erythrocyte distribution width (%), according to either low or high social rank (i.e., LSR and HSR) in Dorper rams (*n* = 24) in northern Mexico (25° N). ^a,b,c^ Least squares means lacking a mutual superscript within response variable differ (*p* < 0.05).

The average values for leukocytes (LEUKO), erythrocytes (ERYTH), hematocrit (HT), mean corpuscular hemoglobin (MCHB), mean corpuscular hemoglobin concentration (MCHBC), and erythrocyte distribution width (EDW), according to social rank and time, are shown in Table 7/Figure 4. Interestingly, while the response variables of LEUKO, ERYTH, HT, MCHB, MCHBC, and EDW were affected by the social rank *x* time interaction, the variables HB and MCV were not affected by said interaction and therefore are not included in Table 7. The variables LEUKO, MCHB, and MCHBC exhibited decreasing values by the end of the experimental period (i.e., Sept). When comparing the initial values (i.e., July) to the final values (i.e., Sept), the plasma levels of LEUKO decreased as the experimental period progressed, which was associated with a reduction in the number of light hours (i.e., photoperiod).

## 4. Discussion

The hypothesis proposed at the start of the study specified that rams with a high rank in the social hierarchy would display better physiological, hematological, and morphometric values, promoting in turn improved indicators regarding sexual behavior (i.e., appetitive and consummatory) and seminal quality, both in the reproductive resting season and at the onset of the reproductive season. Based on the attained results, the working hypothesis is not rejected. Indeed, the number of approaches, anogenital sniffings, mounting attempts, mounts, and mounts with ejaculation were performed in direct proportion to social rank, both in the resting and reproductive seasons. While the low-social-ranked rams displayed a lower number of sexual behaviors, both appetitive and consummatory, the high-social-ranked rams exhibited a greater number of sexual behaviors, as well as a higher ejaculation volume and higher expression of some key hematological markers. In the first male-to-female interaction (i.e., resting season—July), more than 75% of the LSR rams failed to display any sexual activity. These results confirm that high-social-ranked Dorper rams perform a higher number of aggressive behaviors, displacing low-social-ranked rams when exposed to estrus ewes during the male sexual resting season. Moreover, the HSR rams displayed not only a higher seminal quality but also more consummatory sexual behaviors associated with a lower leukocyte population. Interestingly, no differences in live weight were observed between males of high or low social ranking; the main differing physiological variable between social ranks was the male body condition score, which was higher in the high-social-ranked rams.

High-social-ranked males displayed a greater number of threatening behaviors associated with greater sexual appetitive and consummatory actions [11]. As previously reported in Corriedale *x* Milchschaf rams, an increase in sexual appetitive and consummatory behaviors may be linked to the fact that threatening behaviors raise the voluntary feed intake [20]. This suggests that the social interactions come to pass throughout the year irrespective of seasonal variations [20]. A positive relationship between body condition score and sexual appetitive and consummatory behaviors was also reported in adult male Saint Croix sheep [21], which were able to adapt to the disadvantageous environmental conditions of a semi-arid ecotype, while maintaining sexual activity throughout the year. Yet, scrotal circumference was not affected by body condition [21], and neither was it in our study. Moreover, in Malpura adult males, a triple-purpose Indian sheep breed, high-BCS rams (i.e., 3 to 4 units), displayed a greater number of appetitive and consummatory sexual behaviors compared to the low-BCS rams (i.e., 2.5 units) [22]. Moreover, our results are aligned with other studies stating that low BCS reduced the rams’ reproductive performance [23].

When evaluating the impact of body condition in small ruminants [17], a study found that the older the male (i.e., >7 year), the faster his loss of body fat. HSR males always displaced those with a low social rank, affording them privileged access to food and shelter. LSR rams generally have a diminished BCS. Moreover, to preserve reproductive function, productive capacity, and health, males must have suitable amounts of physical assets; the ideal BCS for optimized sexual performance ranges from 2.5 to 3.5 units [17]. The above physiological scenarios agree with the performance of the HSR males in our study; they were of a young age (3 ± 0.5 year), had a high BCS (i.e., 2.66 ± 0.13), and displayed high aggressiveness and numbers of appetitive and consummatory sexual behaviors, which were positively associated with an increased BCS, so they won the sexual–reproductive contest.

The link between consummatory sexual behaviors and indicators of seminal quality throughout the year was addressed in Beni Arouss rams in northern Morocco, with a photoperiod of 14:45 h and 10:00 h in the summer and winter solstices, revealing that the higher the number of sexual behaviors, the greater the seminal quality [24]. Interestingly, this photoperiod is quite similar to that occurring in the location of the present study (i.e., 13:41 h summer solstice and 10:19 h winter solstice), and the photoperiod is the most important reproductive seasonality regulator [10]. Moreover, quite remarkably, the HSR rams performed a greater number of mounts with ejaculation but had a lower latency to mate, as compared to the LSR rams when exposed to an estrus female during the sexual transition period (i.e., the last days of summer). Moreover, the ejaculated volume from the HSR rams was higher than that of the LSR rams. These results confirm a positive relationship between consummatory sexual behaviors and some key seminal quality indicators in the HSR rams in our study. These seminal quality indicators agree with the observed results in Red Sokoto bucks in Nigeria [25], namely, an improved BCS and larger seminal ejaculated volume. Interestingly, in that study, sperm motility and concentration were not affected by BCS, as in our study. In addition, higher plasma levels of testosterone and cortisol have been positively linked to high-BCS rams (i.e., >3.0) in contrast to low-BCS rams (i.e., <2.5) [22]. Although we were not able to quantify plasma testosterone, the previous study helps to explain a possible scenario indicated by the HSR rams in our study. Testosterone concentration is an excellent indicator of seminal production and quality, while spermatogenic activity is directly influenced by the level of testosterone released by the Leydig cells, the testicular endocrine component, modulating in turn the function of Sertoli cells, the gametogenic component of the testes [26].

Our results also confirm that the social rank of Dorper males has significant effects on diverse blood plasma components. All the blood plasma components evaluated were within the normal values established for sheep [27]. Remarkably, the MCHB (pg) and MCHBC (g/dL) values were directly related to social rank. Nevertheless, MCHB and MCHBC did not differ between HSR and LSR rams in the resting reproductive season (i.e., July); however, those plasmatic components were higher in HSR than LSR rams during the transition reproductive period (i.e., Sept). We suggest that this difference was not due to a nutritional deficiency, as both experimental groups received the same diet that covered their nutritional requirements; but, in an intriguing fashion, the HSR rams had not only the best hemogram values but also the highest BCS. Previously, a high value of both the MCHB and MCHBC was reported in multiparous criollo goats, whose diet covered their nutritional requirements, with lower values in those that received a lower level of nutrition [28].

In our study, a social rank *x* time interaction affected the profile of some plasma blood components. Interestingly, the LEUKO (×10⁹/L) concentration of both HSR and LSR rams decreased from July to September; a decreased leukocyte number is linked to a better immune and health status. A positive influence of the photoperiod on growth hormone and leptin concentrations in sheep fed *ad libitum* has been recently reported [29]. Moreover, nutritional supplementation increases the ability of male goats to stimulate and impregnate seasonal anestrous females [30]. In peripubertal male goats, a positive relationship was observed between sexual behavior, semen quality, and erythrocyte population and a leukocyte decrease. Such a scenario activated the adenylate cyclase system that controls testicular testosterone synthesis, while causing a positive stimulating effect on the leukocyte antioxidant defense enzyme system as well as on the spermatogenesis process [31]. It is important to remember that in our study the HSR males also performed a greater number of mounts with ejaculation. Combining these results, we suggest that the complete blood count (i.e., hemogram) when selecting breeding males could be a complementary biomarker, along with routine semen analysis, in order to determine, based on their genetic soundness, the best sires to be mated. Regarding HB (g/dL), a non-significant SR *x* T interaction was observed, with no differences between social rank across time, which was in line with a study on Santa Inés sheep in Brazil [32]. The above results are favorable, since all males showed erythrocyte levels within the normal ranges for sheep, according to their age and gender; thus, not only can a state of anemia be ruled out but a better immune status and therefore well-being status can be assumed. While some health factors directly affect BCS, especially metabolic status and health issues [17], reproductive fitness is substantially modulated by BCS [17,33,34].

Therefore, at this point, a central question emerges: How can we explain the alignment among high social rank, improved BCS, increased appetitive and consummatory sexual activity, and a large ejaculated seminal volume, but not high live weight in rams? There is no easy answer, but allow us to note some interesting facts. When evaluating the BCS effect on hormonal and metabolic markers in Serra da Estrela sheep, a positive correlation between BCS and metabolic status was proposed. Ewes with low nutritional levels (i.e., BCS = 1.25 to 2.0) generated small concentrations of albumin, IGF-1, globulins, and plasma glucose, as well as serum insulin, thyroxine, and triiodothyronine. In contrast, high-BCS ewes (i.e., >3.0) exhibited a more balanced metabolic status, revealing a better nutritional status [35]. Similar results regarding low concentrations of IGF-1 have been described in animals with a low BCS (i.e., <2.0; scale 1 to 5) [36]. Furthermore, undernutrition has shown to depress the hypothalamic–pituitary axis, characterized by hypoglycemia, hypoinsulinemia, and hypoleptinemia, with corresponding higher levels of non-esterified fatty acids (NEFAs), β-hydroxybutyrate, growth hormone, and urea. These alterations induce failures in the secretion of gonadotropins (i.e., LH and FSH), engendering a depressed reproductive performance in small ruminants [37,38]. Moreover, in animals facing an energy shortfall, gonadotropin release declines due to insufficient glucose supply. IGF-I has been shown to be an important intermediary molecule regulating paracrine and/or autocrine processes at the cellular level, including glucose uptake [39], and also enhancing the release of GnRH and LH at the pituitary and hypothalamus levels [38,39].

Additionally, a high BCS in bovines has been positively associated with a boosted metabolic status activating a complex biochemical interplay among diverse metabolic cues along with neuroendocrine signals within the hypothalamic–pituitary–testicular axis, optimizing testicular development through an increased metabolic activity in both Leydig (i.e., steroidogenesis) and Sertoli (i.e., spermatogenesis) cells [40]. Likewise, an increased BCS directly affects GnRH pulsatility, ultimately leading to an increased LH pulse frequency which parallels a crucial increase in testosterone synthesis and secretion [41,42]. Interestingly, not only social dominance but testosterone concentrations and aggressiveness have been strongly associated with widely distributed testosterone receptors at the hypothalamic level [43]. Furthermore, and from a central molecular point of view, in males with a better metabolic status and BCS, four particular genes were upregulated: (1) *SIRT1*, involved in cellular metabolism, (2) *EBP*, related to cholesterol biosynthesis, (3) *INSL3*, linked to testes development, and (4) *CLDN*, aligned to Sertoli cell development [44]. Recently, the possible role of specific genotypes of the melatonin receptor 1A (i.e., *MTNR1A*) gene in social dominance and reproductive success was tested in rams. Although some males carrying certain genotypes of the *MTNR1A* gene displayed the best sexual performance, such superiority was exerted in a non-social hierarchized fashion [45].

The central findings of these previous studies need to be combined with our results with the aim to delineate a conceivable scenario that would support clarification of our research findings. Unquestionably, the HSR rams exhibited the highest BCS—intimately related to body adipose reserves—and the strongest sexual response with more sexual behaviors, appetitive or consummatory, as well as higher seminal quality indicators (i.e., VOL) when mating with sexually active females. Moreover, they also had a reduced leukocyte population. Thus, merging all these results invites us to propose that the HSR rams, through a BCS-dependent effect, may have upregulated some genes (i.e., *SIRT1, EBP, INSL3,* and *CLDN)*, helping to activate different and complex neuroendocrine and metabolic circuitry along with the hypothalamic–hypophyseal–testicular axis. In turn, this may have promoted the reproductive results observed in the HSR rams, though not dependent on live weight. Undoubtedly, some metabolic cues such as blood metabolites (i.e., glucose) and metabolic hormones (i.e., insulin, T3, and T4) and some reproductive hormones (i.e., testosterone) were involved in the physiological and behavioral situation observed in the HRS rams. However, the action or involvement of this situation in the HSR rams, although persuasive, remains to be scientifically proved.

## 5. Conclusions

Our results show that high-social-ranked Dorper rams kept under an intensive management system display agonistic behaviors such as threats, hitting, and pushing against low-social-ranked rams, engendering an increased body condition score (BCS). In turn, this improved BCS positively affected both appetitive and consummatory sexual behaviors (i.e., approaches, mount attempts, anogenital sniffing, mating with ejaculation) and caused both decreased rejections to mount and a reduced latency to ejaculation in the HRS rams, the latter linked to an increased ejaculated seminal volume. No major differences between social status were observed regarding most of the morphometric variables and the hemogram components, except for a reduced leukocyte count in the HSR rams. Knowing a ram’s social rank can be a useful tool to select better rams based on their sexual behavior and seminal quality. Undoubtedly, we have fragmentary knowledge about the precise effect that social rank, BCS, sexual behavior, and hemogram profile have on reproductive success. Nonetheless, our results contribute to a better understanding of how social dominance, aligned with BCS and appetitive and consummatory sexual behaviors, can be associated with, regulate, and even govern out-of-season reproductive success. Future studies should focus on unveiling the multifaceted interplay among blood metabolites, metabolic cues, and reproductive hormones in differentially social-ranked rams.

## Figures and Tables

**Table 1:** Least square means ± standard error for the male-to-male interaction at the onset of the experimental period (July, reproductive arrest), considering threats, hitting, and pushing according to either low (*n* = 15) or high (*n* = 9) social rank in Dorper rams (*n* = 24) in northern Mexico (25° N).

	Social Rank		
	Low ^1^	High ^1^	*p*-Value
Threats (*n*)	0.86 ± 0.5 ^b^	3.22 ± 0.7 ^a^	0.011
Hitting (*n*)	11.8 ± 3.3 ^a^	18.55 ± 4.3 ^a^	0.229
Pushing (*n*)	5.4 ± 1.7 ^a^	8 ± 2.2 ^a^	0.358

^a,b^ Least square means lacking a mutual superscript within response variable (i.e., lines) differ (*p* < 0.05). ^1^ A behavioral study was carried out in July to define the social ranks, either low (LSR) or high (HSR).

**Table 2:** Least square means ± standard error for live weight (LW), body condition score (BCS), scrotal circumference (SCRC), height at the withers (HEIG), length (LENG), and thoracic perimeter (PERI), according to either low (*n* = 15) or high (*n* = 9) social rank in Dorper rams (*n* = 24) in northern Mexico (25° N).

Variables	Social Rank		
	Low ^1^	High ^1^	*p*-Value
LW (kg)	58.71 ± 2.15 ^a^	63.88 ± 2.69 ^a^	0.148
BCS (u)	2.25 ± 0.1 ^b^	2.66 ± 0.13 ^a^	0.024
SCRC (cm)	30.78 ± 0.65 ^a^	32.66 ± 0.81 ^a^	0.087
HEIG (cm)	73.92 ± 1.04 ^a^	76.44 ± 1.30 ^a^	0.148
LENG (cm)	106.14 ± 2.16 ^a^	111.55 ± 2.70 ^a^	0.133
PERI (cm)	107.57 ± 2.12 ^a^	108.77 ± 2.64 ^a^	0.725

^a,b^ Least square means lacking a mutual superscript within response variable (i.e., lines) differ (*p* < 0.05). ^1^ A behavioral study was carried out in July to define the social ranks, either low (LSR) or high (HSR).

**Table 3:** Least square means ± standard error of single effects for the sexual male-to-female interaction considering the variables of rejection to mate, approaches, flehmen, anogenital sniffing, and mounting, according to time (T; i.e., reproductive arrest—July or early reproductive season—Sept) and either low (*n* = 15) or high (*n* = 9) social rank in Dorper rams (*n* = 24) in northern Mexico (25° N) ^1,2^.

	Social Rank (SR)		Time (T)			*p*-Value	
	Low ^1^	High ^1^	July	Sept	s.e. ^2^	SR	T
*Indicator for sexual inactivity*							
Rejection to mate (%)	75 ^a^	0.0 ^b^	39.67 ^a^	35.32 ^a^	0.07	0.0001	0.0007
*Appetitive sexual behavior*							
Approaches (*n*)	0.78 ^b^	3.94 ^a^	3.82 ^a^	0.90 ^b^	0.4	0.0001	0.0001
Flehmen (*n*)	0.82 ^a^	1.05 ^a^	1.59 ^a^	0.28 ^b^	0.2	0.004	0.0001
Sniffings (*n*)	2.07 ^a^	5.0 ^a^	5.62 ^a^	1.44 ^b^	0.6	0.0059	0.0001
*Consummatory sexual behavior*							
Mount with ejaculation (*n*)	0.25 ^b^	2.55 ^a^	1.59 ^a^	1.2 ^a^	0.1	0.0001	0.0001

^a,b^ Least square means lacking a mutual superscript within response variable (i.e., lines) differ (*p* < 0.05). ^1^ A behavioral study was carried out in July to define the social ranks, either low (LSR) or high (HSR). ^2^ Most conservative standard error is presented.

**Table 4:** Least square means ± standard error for the sexual male-to-female actions considering rejection to mate, approaches, flehmen, anogenital sniffing, and mounting, according to time (T; i.e., reproductive arrest—July or early reproductive season—Sept) and either low (*n* = 15) or high (*n* = 9) social rank in Dorper rams (*n* = 24) in northern Mexico (25° N) ^1,2^.

	July		Sept			*p*-Value
	LSR ^1^	HSR ^1^	LSR	HSR	s.e. ^2^	SR × T
*Indicator for sexual inactivity*						
Rejection to mount (%)	78.57 ^a^	0.0 ^b^	57.14 ^a^	0.0 ^b^	0.09	0.0001
*Appetitive sexual behavior*						
Approaches (*n*)	1.5 ^b^	6.55 ^a^	0.07 ^b^	1.33 ^b^	0.69	0.0001
Flehmen (*n*)	1.35 ^ab^	1.88 ^a^	0.28 ^bc^	0.22 ^c^	0.35	0.0001
Sniffings (*n*)	3.78 ^b^	7.66 ^a^	0.35 ^c^	2.33 ^bc^	1.06	0.0001
*Consummatory sexual behavior*						
Mounting with ejaculation (*n*)	0.28 ^c^	3.0 ^a^	0.21 ^c^	2.11 ^b^	0.26	0.0001

^a,b,c^ Least squares means lacking a mutual superscript within response variable (i.e., lines) differ (*p* < 0.05). ^1^ A behavioral test was carried out in July to define the social ranks, either low (LSR) or high (HSR). ^2^ Most conservative standard error is presented.

**Table 5:** Least square means ± standard error for the male-to-female actions during the early reproductive season (i.e., Sept) considering the variables of mating with ejaculation, latency to ejaculation, sperm concentration, mass motility, and ejaculate volume, according to either low (*n* = 15) or high (*n* = 9) social rank in Dorper rams (*n* = 24) in northern Mexico (25° N) ^1^.

	Social Rank		
	Low ^1^	High ^1^	*p*-Value
*Consummatory sexual behavior*			
Mating with ejaculation (%)	42.86 ± 13.0 ^b^	77.78 ± 16.2 ^a^	0.001
Latency to ejaculation (s)	143.07 ± 14.2 ^a^	16.66 ± 17.7 ^b^	0.001
*Indicators for seminal quality*			
Concentration (×10⁶/mL)	1879.5 ± 581.6 ^a^	3471.44 ± 725.4 ^a^	0.10
Mass motility (u)	1.5 ± 0.6 ^a^	2.44 ± 0.7 ^a^	0.33
Ejaculate volume (mL)	0.23 ± 0.1 ^b^	0.57 ± 0.1 ^a^	0.04

^a,b^ Least square means lacking a mutual superscript within response variable (i.e., lines) differ (*p* < 0.05). ^1^ A behavioral study was carried out in July to define the social ranks, either low (LSR) or high (HSR).

**Table 6:** Least square means ± standard error according to social rank (SR) and time (T) (i.e., single effects) for the hemogram response variables of erythrocytes (ERYTH), hemoglobin (HB), hematocrit (HT), mean corpuscular volume (MCV), and erythrocyte distribution width (EDW) in Dorper rams (*n* = 24) in northern Mexico (25° N) ^1,2^.

Variables	Social Rank (SR)		Time (T)				*p*-Value	
	Low ^1^	High ^1^	July	Aug	Sept	s.e. ^2^	SR	T
ERYTH (×10^12^/L)	3.2 ^a^	2.9 ^a^	3.2 ^a^	2.8 ^a^	3.2 ^a^	0.1	0.1	0.17
HB (g/dL)	12.6 ^a^	12.2 ^a^	12.3 ^a^	12.5 ^a^	12.2 ^a^	0.2	0.26	0.75
HT (%)	12.8 ^a^	11.7 ^a^	12.8 ^a^	11.2 ^a^	12.7 ^a^	0.6	0.14	0.15
MCV (fL)	39.4 ^a^	39.3 ^a^	39.3 ^a^	39.1 ^a^	39.7 ^a^	0.2	0.65	0.19
EDW (%)	14.4 ^a^	14.7 ^a^	14.4 ^a^	14.7 ^a^	14.7 ^a^	0.1	0.13	0.25

^a^ Least square means within response variable (i.e., lines) did not differ (*p* < 0.05). ^1^ A behavioral study was carried out in July to define the social ranks, either low (LSR) or high (HSR). ^2^ Most conservative standard error is presented.

**Table 7:** Least square means ± standard error for the interaction of social rank (i.e., LSR and HSR) by time (i.e., 3 dates) for the plasmatic components, leucocytes (LEUKO), erythrocytes (ERYTH), hematocrit (HT), mean corpuscular hemoglobin (MCHB), mean corpuscular hemoglobin concentration (MCHBC), and erythrocyte distribution width (EDW) in Dorper rams (*n* = 24) in northern Mexico (25° N) ^1,2^.

Variables	July		Aug		Sept			*p*-Value
	LSR ^1^	HSR ^1^	LSR	HSR	LSR	HSR	s.e. ^2^	SR × T
LEUKO (×10⁹/L)	82.6 ^a^	82.55 ^a^	84.2 ^a^	79.3 ^ab^	72.6 ^b^	62.6 ^c^	2.8	0.001
ERYTH (×10^12^/L)	3.1 ^ab^	3.5 ^a^	3.2 ^a^	2.4 ^b^	3.4 ^a^	2.9 ^ab^	0.2	0.05
HT (%)	12.6 ^ab^	13.6 ^a^	12.5 ^ab^	9.5 ^b^	13.5 ^a^	11.8 ^ab^	0.9	0.15
MCHB (pg)	39.6 ^bc^	38.6 ^bc^	38.6 ^bc^	51.2 ^a^	37.3 ^c^	45.2 ^ab^	2.0	0.001
MCHBC (g/dL)	101.5 ^bc^	98.8 ^bc^	99.2 ^bc^	132.5 ^a^	94.6 ^c^	116.2 ^ab^	5.7	0.001
EDW (%)	14.2 ^b^	14.6 ^ab^	14.7 ^ab^	14.6 ^ab^	14.5 ^ab^	14.9 ^a^	0.2	0.360

^a,b,c^ Least square means lacking a mutual superscript within response variable (i.e., lines) differ (*p* < 0.05). ^1^ A behavioral study was carried out to define the social ranks, either low (LSR) or high (HSR). ^2^ Most conservative standard error is presented.

## Data Availability

The datasets used in this research are available from the corresponding author on reasonable request.
